# The cranial gland system of *Nasonia* spp.: a link between chemical ecology, evo-devo, and descriptive taxonomy (Hymenoptera: Chalcidoidea)

**DOI:** 10.1093/jisesa/ieaf034

**Published:** 2025-04-15

**Authors:** Holly A Hoag, Monique Raymond, Jonah M Ulmer, Szabina Schwéger, Thomas van de Kamp, Elias Hamann, Marcus Zuber, John H Werren, Grace Gaucher, Missy Hazen, István Mikó

**Affiliations:** Department of Biological Sciences, University of New Hampshire, Durham, NH, USA; Boston IVF, Waltham, MA, USA; Department of Biological Sciences, University of New Hampshire, Durham, NH, USA; USDA-ARS, Washington, DC, USA; National Food Chain Safety Office, Budapest, Hungary; Karlsruhe Institute of Technology (KIT), Institute for Photon Science and Synchrotron Radiation (IPS), Eggenstein-Leopoldshafen, Germany; Karlsruhe Institute of Technology (KIT), Laboratory for Applications of Synchrotron Radiation (LAS), Karlsruhe, Germany; Karlsruhe Institute of Technology (KIT), Institute for Photon Science and Synchrotron Radiation (IPS), Eggenstein-Leopoldshafen, Germany; Karlsruhe Institute of Technology (KIT), Institute for Photon Science and Synchrotron Radiation (IPS), Eggenstein-Leopoldshafen, Germany; Department of Biology, University of Rochester, Rochester, NY, USA; Department of Biological Sciences, University of New Hampshire, Durham, NH, USA; Department of Biological Sciences, University of New Hampshire, Durham, NH, USA; Huck Institute of the Life Sciences, Pennsylvania State University, University Park, PA, USA; Department of Biological Sciences, University of New Hampshire, Durham, NH, USA

**Keywords:** SBF-SEM, craniofacial, parasitoid, speciation, mandibular rods

## Abstract

*Nasonia* is an emerging model system for investigating the evolution of complex species-specific behavioral and morphological phenotypes. For example, the male head shape differs considerably between *Nasonia* Ashmead (Hymenoptera: Chalcidoidea) species. In addition, differences in courtship behaviors, and possibly influences of a male-specific aphrodisiac pheromone, contribute to interspecific prezygotic isolation. However, the possible relationships between courtship, pheromone signaling, and male head shape are unknown. Using multimodal imaging techniques, we conducted a comprehensive examination of the skeletomuscular and exocrine gland systems of the lower head region of all 4 *Nasonia* species and their sister genus *Trichomalopsis* Crawford (Hymenoptera: Chalcidoidea). This analysis reveals the presence of 3 undescribed exocrine glands in the lower head region and a unique mandibular modification, the basal mandibular carina, that might be involved in pheromone spread. We performed morphometric and volumetric analyses using 3D datasets from synchrotron X-ray microtomography and found that the size of the genomandibular gland and the corresponding basal mandibular carina correlates with both interspecific courtship length and head shape differences, indicating that this gland is a likely source of the oral aphrodisiac pheromone. These differences correlate with the prevalence of within-host mating rather than phylogenetic relatedness in *Nasonia* species, with increased within-host mating occurring in species with larger genomandibular glands. Our findings create an opportunity to better understand the complex gene regulatory networks underlying superficially unrelated traits and serve as a link between behavior, chemical ecology, evo-devo, and descriptive taxonomy.

## Introduction

Selection for traits that facilitate complex behaviors is also associated with anatomical differences in location, form, and connection, each with respective functional networks (Kaminski et al. 2019). Identifying the specific attributes of anatomical structures provides valuable insight into the mechanisms that drive behavior (Webber et al. 2023), especially in the context of genetic control over anatomy (Koentges 2008). In many animal species, males employ unique courtship behaviors to secure copulation with females (Coulter and Prather 2020). Courtship behaviors are often diverse and complex, occurring in simultaneous sensory modalities (Mitoyen et al. 2019). The study of courtship behavior in model organisms, particularly in insects, which present observable behaviors, have short life cycles, and can be genetically manipulated, has the power to reveal new knowledge of the fundamental biological processes and the principles governing evolutionary adaptation (Yu et al. 2010, Cargnelutti et al. 2023).

For over half a century, research on the model system *Nasonia* Ashmead (Hymenoptera: Chalcidoidea) has fostered a deep understanding of male courtship behavior (Barrass 1960, van den Assem 1973, van den Assem et al. 1980, van den Assem and Werren 1994, Clark et al. 2010, Niehuis et al. 2013, Benetta et al. 2021). *Nasonia* is a benchmark model genus that facilitates the exploration of mechanisms and processes involved in speciation, as it is comprised of multiple closely related species (Niehuis et al. 2013). *Nasonia vitripennis* Walker is cosmopolitan, *Nasonia longicornis* Darling is found in western North America, and both *N. giraulti* Darling and *N. oneida* Raychoudhury et al. occur in eastern North America.

**Fig. 1.**
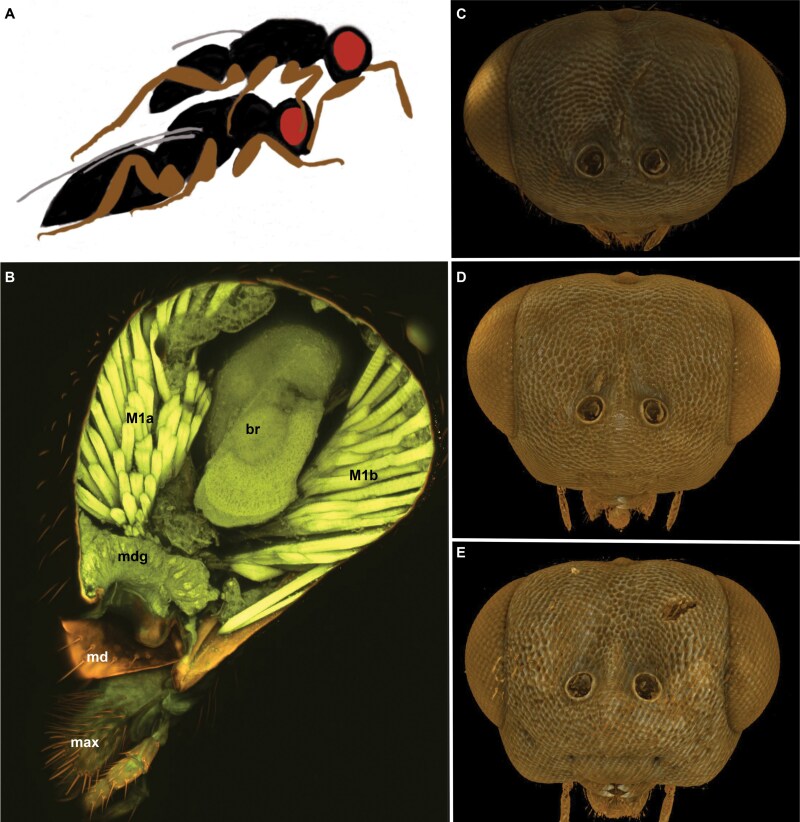
Phenotypic traits related to the aphrodisiac pheromone in *Nasonia* species. **A**, Head nodding during courtship of *Nasonia*. Males touch the female antennae preceding copulation, increasing the sexual desire of the female. **B**, CLSM micrograph of a bisected head of *N. giraulti* showing the enlarged mandibular gland (mdg). **C–E**, Frontal view of male heads of *Nasonia vitripennis* (C), *N. longicornis* (D), and *N. giraulti* (E) showing interspecific differences in gena convexity (mdg = mandibular gland, md = mandible, M1a = mandibular adductor anterior, M1b = mandibular adductor posterior, br = brain, max = maxilla).

*Nasonia* are parasitoid wasps that lay their eggs within the puparium of various fly species (Darling and Werren 1990). The developing wasps devour the host and emerge from the host puparium (van der Merwe 1943, Whiting 1967). Mating occurs either within or outside of the host, with the prevalence of within-host mating (WHM) varying by species (Trienens et al. 2021). Trienens at el. (2021) found that WHM is prominent in *N. giraulti,* whereas *N.vitripennis* mating occurs outside of the host, and both *N. longicornis* and *N. oneida* display an intermediate degree of WHM. *Nasonia* females typically mate once, and interspecific copulations often result in bacterial (Wolbachia) induced reproductive incompatibility (Breeuwer and Werren 1990, Bordenstein et al. 2001) and/or less fit offspring (Breeuwer and Werren 1995, Clark et al. 2010). The species show different levels of premating behavioral discrimination against heterospecific males (Bordenstein et al. 2001, Ruther and Hammerl 2014). In addition, *N. giraulti* and *N. longicornis* can mate within the hosts, which increases inbreeding and reduces opportunities for heterospecific matings. Courtship behavior also provides an opportunistic mechanism for female choice (Trienens et al. 2021). Males, while mounted on a female, initiate courtship with a sequence of rhythmic “head-nods” and wing vibrations following a circadian rhythm (Barrass 1960, van den Assem et al. 1980, 1981, van den Assem and Werren 1994, Clark et al. 2010). Crucially, the first head nod in each series is correlated to the induction of female receptivity (Fig. 1A, van den Assem et al. 1980). Females signal their receptivity by lowering their antennae and protruding their genitalia, or they reject the male, leading to dismount and the cessation of courtship (Barras 1960). Ruther et al. (2010) provides videographic documentation of the unique *Nasonia* courtship style.

The intensity of nodding in courtship cycles is species-specific: the courtship cycle of *Nasonia longicornis* is approximately twice as long in duration as that of *N. vitripennis*; *Nasonia giraulti* has a cycle about 3 times longer than *N. vitripennis* and displays a significant increase in the number of head-nods per series (van den Assem and Werren 1994). Heterospecific discrimination by female *Nasonia* may be associated with variable factors related to the male aphrodisiac pheromone as well (Diao et al. 2016). Although the exact chemical composition and origin of the aphrodisiac remains unknown, it has been suggested that an exocrine gland in the lower head region, such as a mandibular gland (Fig. 1B) is a potential source (van den Assem et al. 1980, 1981, Mikó and Deans 2014).

The shape of the lower head region is also used for diagnostic purposes to differentiate *Nasonia vitripennis*, *N. giraulti,* and *N. longicornis* (Darling and Werren 1990). The convexity of the gena, the cuticular region between the base of the mandible and the compound eye, is sexually dimorphic and shows quantifiable species-level differences that correlate with variations in courtship intensity. In *N. vitripennis*, the gena of males is comparable to that of conspecific females, being small and minimally convex. As the lineage progresses to *Nasonia longicornis* and then *N. giraulti*, the gena becomes more convex (*N. oneida* gena convexity has previously not been assessed) (Fig. 1C–E, Werren et al. 2016). Dramatic differences and abnormalities can be found in the head shapes of F2 hybrid haploid males (Werren et al. 2016). This variation, including genal morphology in *Nasonia* species, was employed to explore the evolution of complex genetic traits and to investigate epistatic interactions underlying craniofacial development and disease (Werren et al. 2016). Cohen et al. (2021) hypothesized that differences in genal convexity might be related to the size of the mandibular gland. Exploration of the lower head gland, a possible connection between cranial morphometry and variations in courtship behavior can contribute to this knowledge base, adding a new element of biological understanding.

This study, employing confocal laser scanning microscopy, synchrotron X-ray microtomography, and serial block-face scanning electron microscopy, uses the *Nasonia* model system to reveal a previously overlooked connection between chemical ecology, behavioral genetics, and evolutionary development.

## Materials and Methods

Virgin males and females used in this research were collected and sexed in the immobile black pupal stage by cracking open their host fly puparia 1 to 2 d before eclosion. Sex identification was performed by looking for the presence of the ovipositor and differences in leg coloration. Specimens were fixed after eclosion in 95% ethanol or in glutaraldehyde solution. 3D datasets of the wasps were collected using serial block-face scanning electron microscopy (SBF-SEM; 2 male *Nasonia giraulti*), confocal laser scanning microscopy (CLSM), and synchrotron-based micro-CT (SRμCT; 10 male and 10 female *N. vitripennis*, 10 male and 7 female *N. longicornis*, 9 male and 9 female *N. giraulti,* 8 male *N. oneida,* and 10 male *Trichomalopsis sarcophagae* (Gahan)) were used to generate high-resolution TIFF image sequences. Synchrotron X-ray microtomographic data are deposited at the RADAR4KIT repository of Karlsruhe Institute of Technology (https://doi.org/10.35097/98atmbk2tcvuu3s4).

Terminology of the phenotype statements used in the description is mapped to the Hymenoptera Anatomy Ontology (Yoder et al. 2010; available at http://purl.obolibrary.org/obo/hao.owl) using a URI table (Supplementary Table 1., Seltmann et al. 2012). The gena, previously referred to as the cheek (Werren et al. 2016, Cohen et al. 2021), is the region of the cranium between the lower margin of the compound eye and the lateral margin of the oral foramen (hypostoma). Insect exocrine glands are classified based on the anatomy of cells from the single-layer epithelium into 2 classes (Noirot and Quennedey 1974, 1991, class II cells from their original classification (1974) are equivalent to oenocytes). Class I cells are characterized by their microvillus-rich cell membrane facing the cuticle while class III cells are characterized by the microvillus-rich terminal region of the invaginated cell membrane that surrounds the apical region of the cell process of the canal cell (end apparatus).

CLSM images were taken on glycerin-stored specimens dissected with an Olympus SZX16 stereomicroscope with SDFPLAPO 2XPFC objective using a Zeiss LSM 710 Confocal Microscope at the Cellular and Molecular Imaging Facility, NC State University. We used an excitation wavelength of 488 nm and an emission wavelength of 510 to 680 nm, detected using 2 channels and visualized separately with 2 pseudocolors (510 to 580 nm = green; 580 to 680 nm = red).

High-throughput synchrotron X-ray microtomography (SR-μCT) of ethanol-preserved specimens was performed at the Imaging Cluster of the KIT Light Source. We employed a parallel polychromatic X-ray beam produced by a 1.5 T bending magnet spectrally filtered by 0.5 mm aluminum to obtain a peak at about 15 keV. A fast indirect detector system was employed, consisting of a 12 µm LSO:Tb scintillator (Cecilia et al. 2011) and a diffraction-limited optical microscope (Douissard et al. 2012) coupled with a 12-bit pco.dimax high-speed camera with 2016 × 2016 pixels. For each scan, we took 200 dark field images, 200 flat field images, and 3000 equiangular spaced radiographic projections in a range of 180° with 70 frames per second, resulting in a scan duration of about 43 s each. The magnification of the optical system was adjusted to 10×, yielding an effective X-ray pixel size of 1.22 μm (Dos Santos Rolo et al. 2014). The control system concert (Vogelgesang et al. 2016) was employed for automated data acquisition. The tomograms were reconstructed with tofu (Faragó et al. 2022).

For serial block-face scanning electron microscopy (SBF-SEM), specimens were dissected in cacodylate buffer, fixed in glutaraldehyde, and then stained with osmium tetroxide, potassium ferrocyanide, thiocarbohydrazide solution, uranyl acetate, and lead aspartate (Protocol available at https://doi.org/10.6084/m9.figshare.4993796.v1, modified from Deerinck et al. 2010). Following fixation protocol, specimens were dehydrated through an ethanol series and embedded in Eponite. Blocks were trimmed and sectioned using a Leica UCT ultramicrotome, then mounted into a Zeiss SIGMA VP-FESEM with a Gatan 3View2 accessory for sectioning and imaging. Images were aligned and cropped in Fiji (Version 2.0.0, Schindelin et al. 2012).

3D reconstruction of the cranium, mandible, exocrine glands, and mandibular muscles was performed with Fiji using the segmentation editor and ROI manager modules. Every 10th slice of the tomographic volume was segmented and followed by an automated interpolation using Biomedisa (https://biomedisa.info/; Lösel et al. 2020). Volume renderings of the segmented structures and cranium were generated using Drishti (Limaye 2012) and 3D Slicer (Fedorov et al. 2012) using the Slicermorph extension (Rolfe et al. 2021).

Cranial morphometrics (gena height, GH, the longest perpendicular anatomical line between gena width (GW) and ventrolateral genal margin; gena width, distance between the median point on the distal margin of the clypeus and the lowest point of the compound eye, head width, HW, longest distance between the lateral eye margins and height of basal mandibular carina, BCH; Figs. 3A, B, Supplementary Fig. 1A, B, Werren et al. 2016) were measured with Fiji using anterior view screenshots of the 3D volume rendered models from 3D Slicer using the slicermorph extension. The volumes of the subcuticular space (scs: Supplementary Fig. 1C, D) were measured using 3D slicer image segmentations and Quantification/Segment Statistics (Labelmaps statistic) modules. Overall gland volume was impossible to measure because in most cases we were not able to differentiate gland cells from the adjacent fat body tissues (Fig. 7D–H). Volumes were calculated in Microsoft Excel by summing the surface area of each gland outline and then multiplying this value by the slice spacing of 1.22 μm. SCV, FE, BCH were standardized with HW as a body-size proxy (Ohl and Thiele 2007) and analyzed using a Kruskall-Wallis non-parametric ANOVA and Spearman’s rank correlation. Morphometric measurements are available from 10.6084/m9.figshare.28598108.

## Results

### Cuticle

**Fig. 2.**
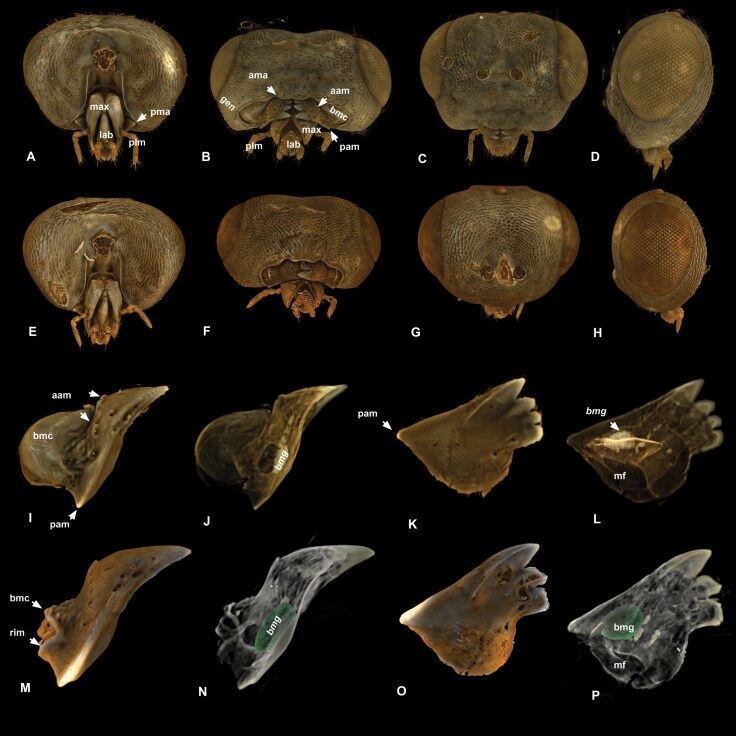
**Volume rendered micrographs (Drishti) showing cuticular anatomical structures of the cranium and mandible and the basimandibular gland of *Nasonia giraulti.*** A–D, cranium, male, E–H, cranium, female. A, E—posterior view, B, F—ventral (oral) view, C, G—anterior view, D, H—lateral view. I–L, mandible, male, M-P, mandible, female. I, M—ventral view, 0 transparency, J, N—ventral view 50% transparency showing basimandibular gland (img), K, O—posterior (internal) view, 0 transparency, L, P—posterior (internal) view, 50% transparency showing basimandibular gland (bmg, aam = anterior angle of the mandible, bmc = basal mandibular carina, lab = labium, max = maxilla, pam = posterior angle of the mandible, plm = maxillary palp).

**Fig. 3.**
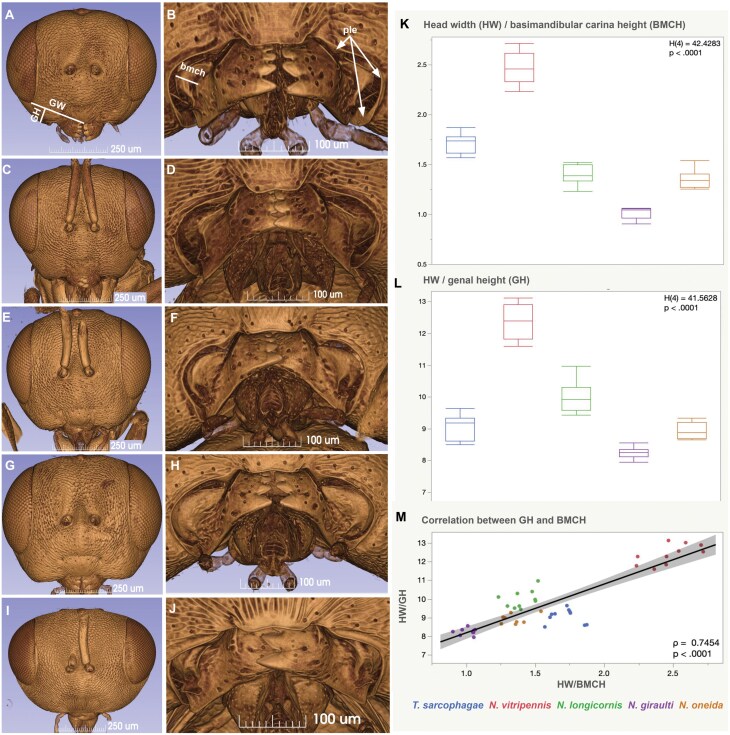
**Species level variability of the cranium and mandible in male Pteromalidae.** A, B, *Nasonia vitripennis.* C, D, *N. longicornis.* E, F, *N. oneida.* G, H, *N. giraulti.* I, J, *Trichomalopsis sarcophagae*. K, BMCH (basimandibular carina heigth) by species, Kruskal-Wallis test: The differences between the rank sums of 425 (*N. vitripennis*), 198 (*N. longicornis*), 45 (*N. giraulti*), 135 (*N. oneida*), and 325 (*T. sarcophagae*) were significant, H(4) = 42.4283, *P* < 0.0001. Post hoc pairwise comparisons using Dunn’s test indicated that BMCH in *N. vitripennis* were observed to be significantly different from those of *N. longicornis* (*P* = 0.0023), *N. giraulti* (*P* < 0.001), and *N. oneida* (*P* = 0.0009). This metric in *T. sarcophagae* was observed to be significantly different from that of *N. giraulti* (*P* = 0.0001). No other differences were statistically significant. L, GH (gena height) by species, Kruskal-Wallis test: The differences between the rank sums of 425 (*N. vitripennis*), 321 (*N. longicornis*), 46 (*N. giraulti*), 142 (*N. oneida*), and 194 (*T. sarcophagae*) were significant, H(4) = 41.5628, *P* < 0.0001. Post hoc pairwise comparisons using Dunn’s test indicated that normalized gena heights in *N. vitripennis* were observed to be significantly different from those of *N. giraulti* (p < 0.001), *N. oneida* (*P* = 0.0015), and *T. sarcophagae* (*P* = 0.0018). This metric in *N. giraulti* was also observed to be significantly different from that of *N. longicornis* (*P* = 0.0002). No other differences were statistically significant. M, HW/BMCH vs HW/GH, Spearman’s correlation: There was a significant positive monotonic relationship between the two metrics, rs(45) = 0.7454, *P* = < 0.0001.

The gena (area between the compound eye and the oral foramen) and the mandibles show both interspecific and sexual differences in *Nasonia* (Figs. 2 and 3). The outline of the gena in anterior view is the most convex in *N. giraulti* males followed by *N. longicornis* and *N. oneida,* which share the same genal convexity and mandible width (Fig. 3C, E, G). *N. vitripennis* and *Trichomalopsis sarcophagae* males (Fig. 3A, I) along with females (Fig. 2E–H) of all species possess the less convex, almost straight gena and short mandible (Figs. 2 and 3).

The mandible is connected to the cranium via the basal mandibular conjunctiva (bmm: Figs. 5B, 7D, 8C). The mandibular site of origin of the basal mandibular conjunctiva corresponds to a slightly elevated rim (rim: Fig. 2M) that surrounds the mandibular foramen (mf: Fig. 2L, P). On the external surface of the mandible, the rim is distinctly set off the proximal mandibular margin and gradually extends into the basal mandibular carina (bmc: Fig. 2I, M).

**Fig. 4.**
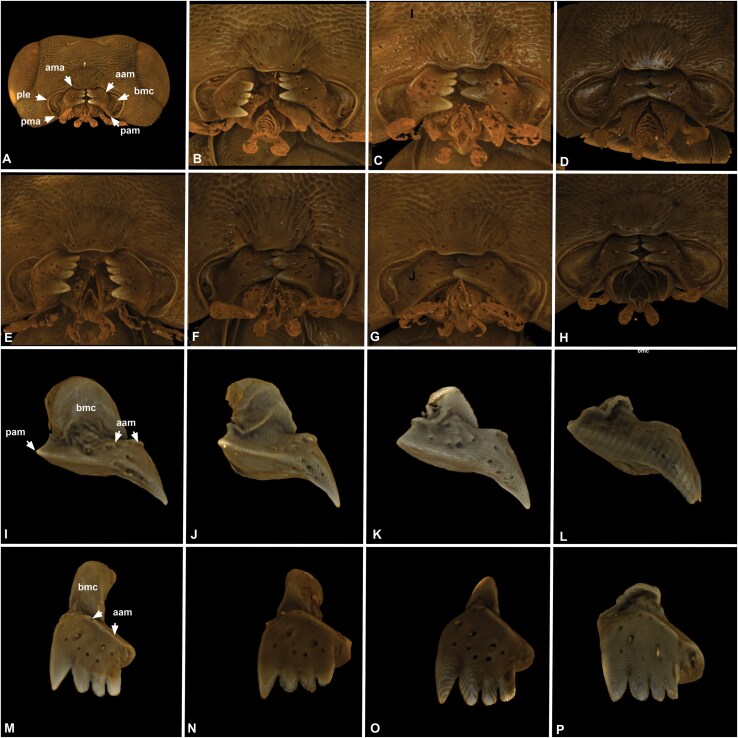
**Volume rendered micrographs showing the mandibles of *Nasonia* specimens.** A–H, The anterior and posterior angles of the mandibles (aam, pam) do not articulate with the cranium at rigid articulations but can slide on the pleurostoma (ple) and move in multiple axes (A–C, E–G, *N. vitripennis*, D, H, *N. giraulti*). I–P, The size of the basal mandibular carina (bmc) relative to the length of the mandible is sexually dimorphic and exhibits species-level variation: it is largest in *Nasonia giraulti*, medium size in *N. longicornis* and *N. oneida*, and smallest in *N. vitripennis*. In females of all species, it is reduced to a low ridge. The anterior and posterior angles of the mandible are neither sexually dimorphic nor species-specific.

The size of the basal mandibular carina is sexually dimorphic, it is shallow and not higher than wide in females and it is at least 3 times as high as wide in males (Fig. 2I–P, N–P). The carina of different species is similar in height and width in female specimens and it has a different height in male specimens of the 4 species (Fig. 4I–P). It is half the width of the mandible in lateral view in *N. vitripennis*, equals the mandible width in *N. longicornis* and *N. oneida,* and is twice as high as the mandible width in *N. giraulti.*.

The mandible has 3 to 4 apical teeth and articulates with the cranium at the anterior and posterior angles of the mandible (aam, pam: Fig. 2B, I). The angles do not articulate with the cranium at rigid articulations but can slide on the pleurostoma and move in multiple axes (ple: Fig. 4A–H).

**Fig. 5.**
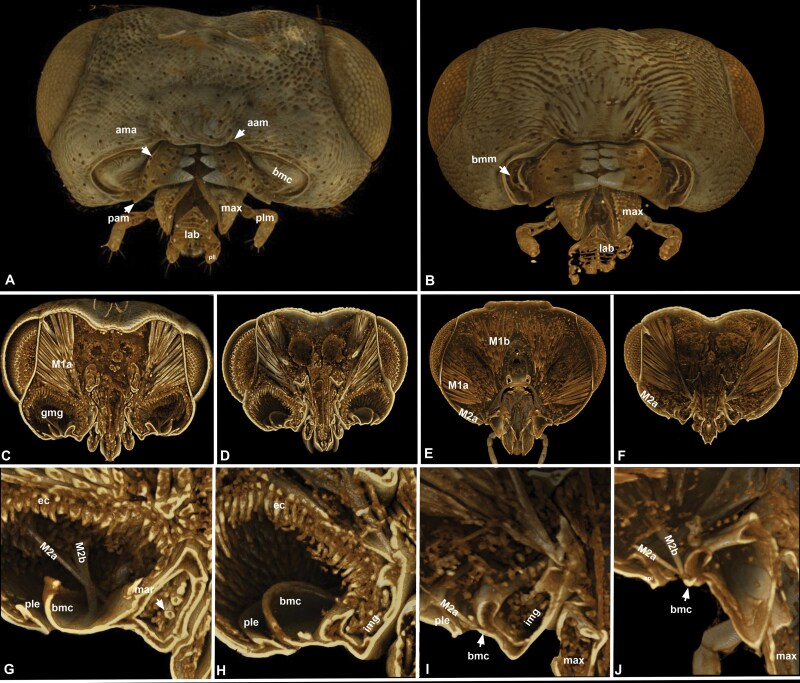
Volume rendered micrographs (Drishti) showing the head and lower cranial regions of *Nasonia giraulti*. A, C, D, G, H, male, B, E, F, I, J, female. A, B, oral view showing the mouthparts, C–J, Sagittal sections of the head showing the epithelial cell layer of the genomandibular gland, the subcuticular space and the adjacent surfaces of the basal mandibular carina and epistoma (aam = anterior angle of the mandible, bmc = basal mandibular carina, ec = epithelium of the genomandibular gland, ple = pleurostoma, gmg=genomandibular gland, lab = labium, M1a, M1b = mandibular adductor muscles, M2a, M2b = mandibular abductor muscles).

When the mandible is fully retracted, the basal mandibular conjunctiva is visible externally in females lateral to the basal mandibular carina but obscured by the cranium in males and the basal mandibular carina fully occupies the oral foramen laterally (bmm: Fig. 5B). The outer surface of the basal mandibular carina and the internal surface of the pleurostoma are adjacent in males and the basal mandibular conjunctiva is enclosed between them (Figs. 7D and 8C). The basal mandibular carina and the pleurostoma are set apart in females and the basal mandibular conjunctiva is not enclosed between them (Fig. 7E).

### Muscles

**Fig. 6.**
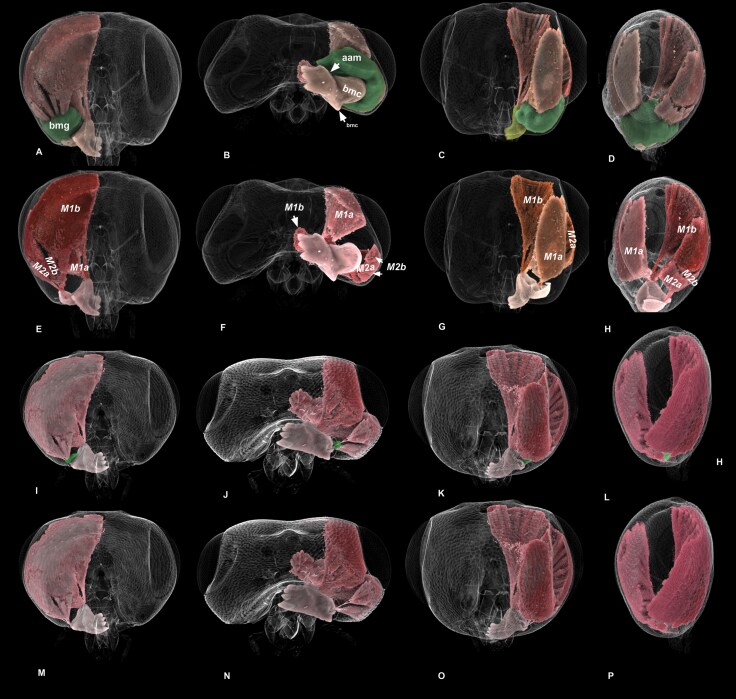
**Volume rendered micrographs (Drishti) showing the mandibular muscles and the genomandibular gland of *Nasonia giraulti*. A–D**, external view, **E–L**, internal view. A, E, I, posterior, B, F, J ventral (oral) view, C, G, K anterior view, D, H, L lateral view. **M–P**, Sagittal sections of the head of *N. giraulti* showing the epithelial cell layer of the genomandibular gland, the subcuticular space and the adjacent surfaces of the basal mandibular carina and epistoma. (aam = anterior angle of the mandible, bmc = basal mandibular carina, ec = epithelium of the genomandibular gland, epi = epistoma, gmg = genomandibular gland, lab = labium, M1a, M1b = mandibular adductor muscles, M2a, M2b = mandibular abductor muscles, max = maxilla, pam = posterior angle of the mandible, pll = labial palp, plm = maxillary palp, scs = subcuticular space of the genomandibular gland).

**Fig. 7.**
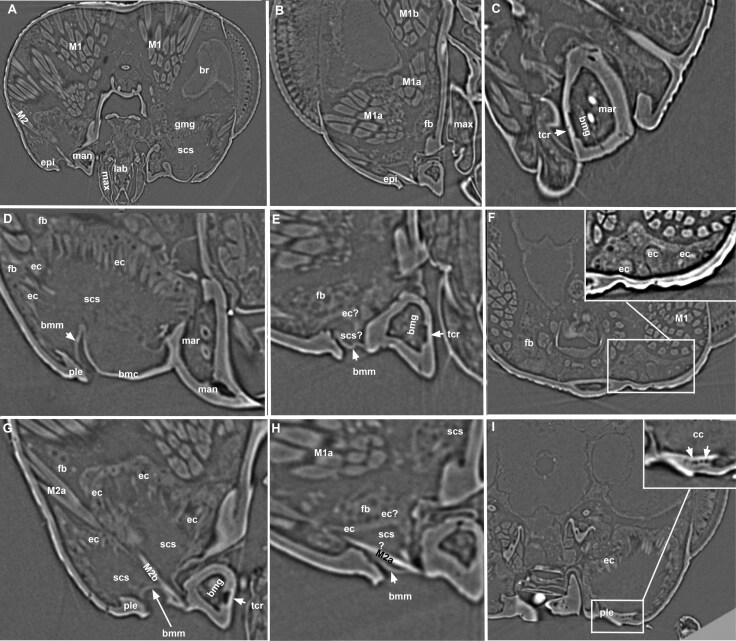
**micro-CT micrographs showing the skeletomuscular and exocrine gland systems of the lower head regions of *Pteromalidae*.** A, D, F, G, I, *Nasonia giraulti* male, B, E, H, *N.giraulti* female, C, *Trichomalopsis sarcophagae* male. Animated GIF version of D is available from 10.6084/m9.figshare.28300592, and animated GIF version of F is available from 10.6084/m9.figshare.28300607. The mandibular abductor muscles (M2a, b) inserts at the base of the basal mandibular carina (bmc). The mandibular rods (mar) are non-glandular and solid invaginations of the mandibular sclerite. The epithelial cell layer of the class I basimandibular gland (bmg) corresponds to a thinner cuticular region of the mandible (tcr). In some cases, it is complicated to separate the cuboidal fat body cells (fb) from the adjacent columnar epithelial cells of the genomandibular gland (ec) in males as the border between them is not always clear (A, D, G). The epithelium of the genomandibular gland is much thinner in females (ec: E, H). The mandible is connected to the pleurostoma (ple) by the basal mandibular conjunctiva (bmm). The subcuticular space of the genomandibular gland develops between the epithelial gland cells (ec) and the cuticle of the pleurostoma (ple), basal mandibular conjunctiva (bmm) and the basal mandibular carina (bmc). Emptying of the gland might be governed by the contraction of the mandibular abductor muscles (M2a, M2b). The epithelial cells of the class III genal gland (ec: F, I) are adjacent to the genal cuticle just ventral to the compound eye and open, via separated cuticular canals (cc), on the pleurostoma (ple).

The mandibular sites of insertions of the 2 adductor and 2 abductor muscles of the mandible are distinctly separated from each other. The anterior and posterior adductor muscles of the mandible (M1a, M1b) insert proximomedially on the mandibular margin (Figs. 5–7). The median (M2b) and lateral (M2a) abductor muscles of the mandible insert posteriorly at the base of the basal mandibular carina (Figs. 5–7) both in females and males.

### Mandibular Rods

**Fig. 8.**
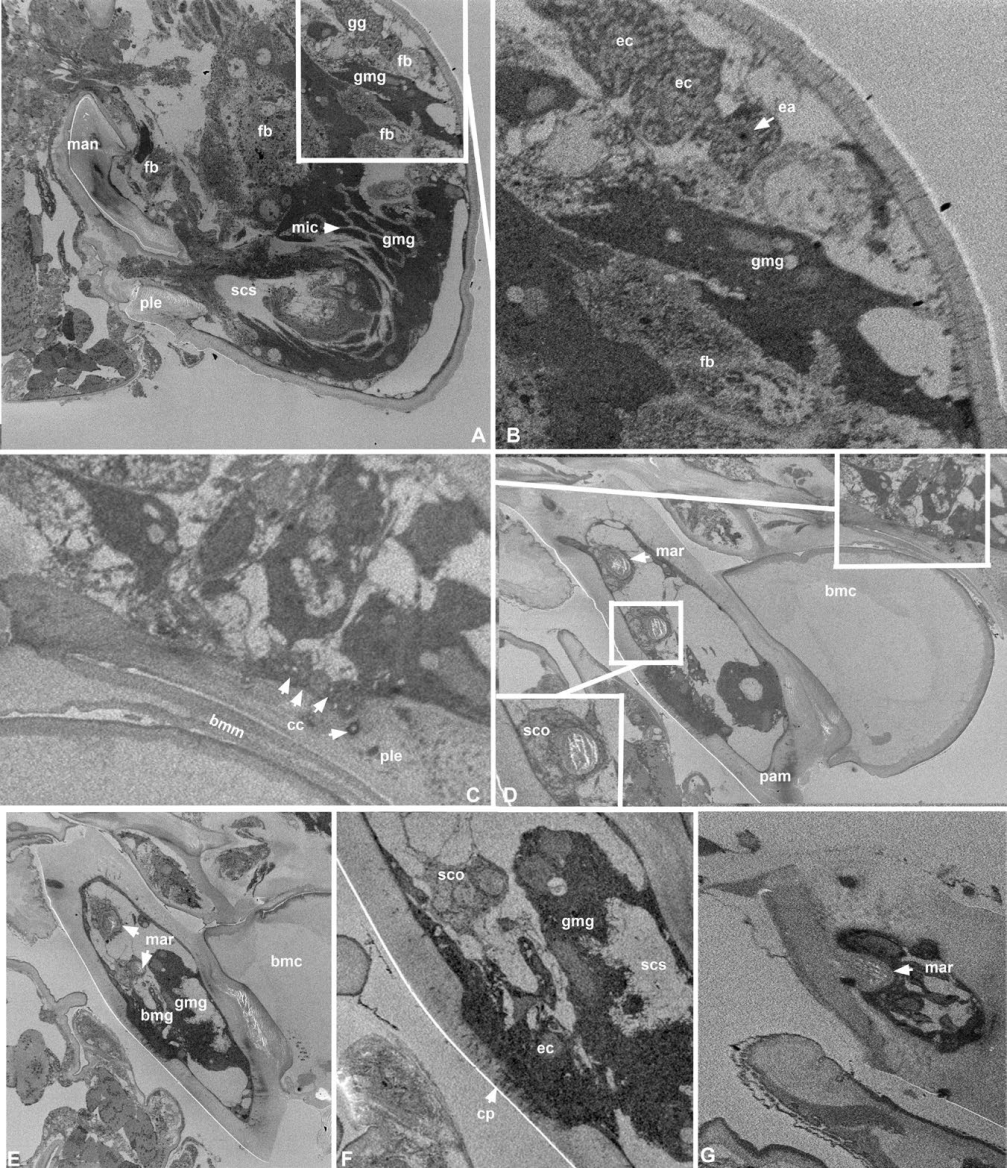
**SBFSEM micrographs of the exocrine gland systems of the lower head region of *Nasonia giraulti* male.** Animated GIF version of A is available from 10.6084/m9.figshare.28300616, B, C from 10.6084/m9.figshare.28300670 and D–G from 10.6084/m9.figshare.28300748. The epithelial cells of the class I genomandibular gland (gmg) are characterized by the electron-dense cytoplasm and the microvilli rich distal cell membrane (mic) and the lack of end apparatuses and cuticular canals (A). Cells of the genal gland (gg) are less electron dense and possess microvilli-embedded end apparati (ea) that continues into cuticular canals (cc) exiting at the pleurostoma (B, C). The mandibular rods (mar) are solid cuticular invaginations that are not associated with any glandular tissue but are connected to cellular structures resembling scolopale cells (sco?). (bmc = basal mandibular carina, bmg = basimandibular gland, bmm = basal mandibular membrane, cc = cuticular ducts of genal gland, ea = end apparatus, ec = epithelial cell of the genomandibular gland, epi = epistoma, fb = fat body, gmg = genomandibular gland, gg = genal gland, man = mandible, mar = mandibular rod, mic = microvilli, scs = subcuticular space, pam = posterior mandibular margin, neu = neuron, nuc = nucleus).

The mandibular rods are solid structures that are continuous distally with the mandibular cuticle close to the tip of the mandibular teeth and do not connect proximally to any sclerite or muscle tissue (mar: Figs. 7C,D, 8D, E, G). We did not locate any cuticular canals or densely pored regions in the rods and adjacent cuticular regions. Six to eight cellular structures with indistinct nuclei and with marginal electron dense particles are connected to the base of each rod (sco: Fig. 8D, F).

### Glandular Epithelia of the Lower Head Region

Two class I (genomandibular and basimandibular gland) and one class III gland (genal gland) are present in the lower head region of *Nasonia* species.

The class I basimandibular gland (bmg) is located at the ventral wall of the mandible (bmg: Fig. 2J, N, L, P). The thickness of the mandibular cuticle abruptly decreases at a smaller region on its ventromedial side where the cuticle is 5 times as thin as at the surrounding regions (tcr: Fig. 7C, E, G). The area with decreased thickness is densely fenestrated by cuticular pores (cp: Fig. 8F) and is adjacent to enlarged, electron-dense epithelial cells with electron-lucent nuclei (ec: Fig. 8F). We did not observe any difference in the size of the basal mandibular gland between male and female specimens or specimens from different species.

The class III genal gland is located anteriorly on the gena and is composed of 7 to 8 enlarged gland cells (gg, ec: Figs. 7F, 8A, B). The cytoplasms of these cells are electron-dense and the cells are filled with white electron-lucent vesicles. Each cell is characterized by the presence of a single microvillus-embedded end apparatus (ea: Fig. 8B) that is continuous with cuticle-embedded ducts that open anterolaterally on the pleurostomal surface without ductule conglomeration (cc, ple: Figs. 7I and 8C). The glandular cells are located between the dorsal genomandibular gland and the anterior branch of the mandibular adductor muscle (Figs. 7F and 8A). We did not observe any difference in the size of the genal glands between male and female specimens or specimens from different species.

The class I genomandibular gland is composed of the epithelial cells of the basal mandibular region, the pleurostoma, and the conjunctiva between the mandible and the pleurostoma. In male specimens, the epithelial cell layer of the genomandibular gland is thicker, composed of columnar cells that are 6 to 7 times as tall as wide and 4 to 5 times as thick as the cuticle of the basal mandibular conjunctiva (ec, bmc: Fig. 5G, H, 7D, G). We were not able to unambigously detect the epithelial cells and subcuticular space in female specimens (ec?, scs?: Fig. 7E, H).

A distinct subcuticular space is developed between the epithelial cell layer and the corresponding cuticle in all male specimens except in *N. vitripennis*. In this species and in all female specimens of all species, the subcuticular space is absent or strongly reduced (scs: Fig. 7E, H). The epithelial cells of the genomandibular gland are more electron-dense than the genal gland cells (gg vs gmg: Fig. 8A, B). We did not observe microvilli-embedded end apparatuses of cuticular ducts inside the cells of the genomandibular gland but the distal margin of the cells are equipped with microvilli rich apical region (mic: Fig. 8A). The cells of these glands are gradually tapering toward the cuticle (ec: Fig.s 5G, H, 7D, G).

We did not observe differences in the thickness of the cell layer between males of the 4 *Nasonia* species, but the cells are 2 to 3 times as large in *T. sarcophagae* than in *Nasonia* spp.

**Fig. 9.**
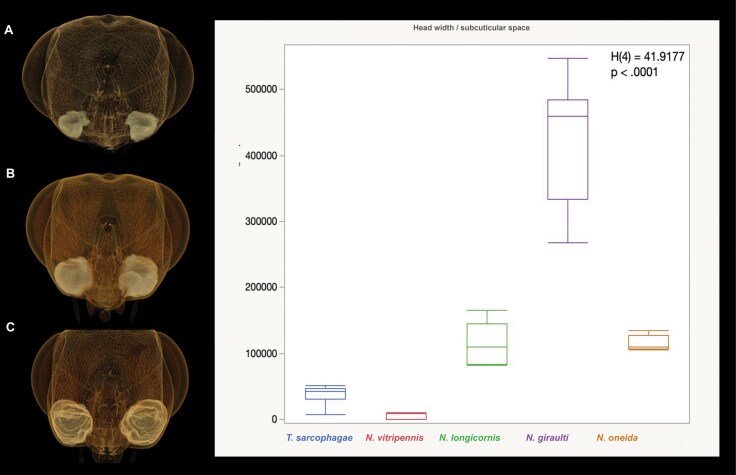
**Variation of the genomandibular gland in *Nasonia* species. A–C.** Volume rendered micrographs (Drishti) showing the variation in mandibular gland size in *Nasonia* species, *N. vitripennis* (A), *N. longicornis* (B), *N. giraulti* (C). The gland size in *N. oneida* (not illustrated) is not different from that of *N. longicornis*. **D.** Kruskal-Wallis: The differences between the rank sums of 58 (N. vitripennis), 294 (N. longicornis), 387 (N. giraulti), 237 (N. oneida), and 152 (T. sarcophagae) were significant, H(4) = 41.9177, *P* = < 0.0001. Post hoc pairwise comparisons using Dunn’s test indicated that normalized subcuticular gland volume in *N. vitripennis* was observed to be significantly different from those of *N. longicornis* (*P* = 0.0013), *N. giraulti* (*P* < 0.001), and *N. oneida* (*P* = 0.0027). This metric in *N. giraulti* was also observed to be significantly different from that of *T. sarcophagae* (*P* = 0.0001). No other differences were statistically significant.

The volume of the subcuticular space in the genomandibular gland (space between the glandular epithelium and the cuticle) has significant differences amongst sexes and amongst the male specimens of different species (Figs. 5, 7, and 9). The volume is the largest in *N. giraulti* males, followed by *N. longicornis* and *N. oneida* males and is smallest in *N. vitripennis* males. For *N. oneida* males the gland volume was similar to that of *N. longicornis*. The gland volume of *T. sarcophagae* males was the second smallest of all 5 species investigated (Fig. 7). The genomandibular gland of female specimens was extremely small and ill-defined, as it was difficult to differentiate between glandular cells and fat bodies. For this reason, the subcuticular space in female specimens was not measured, as it would have been impossible to obtain accurate data. For the purposes of this study, the genomandibular gland in *Nasonia* females is considered to be absent.

## Discussion

Van den Assem et al.’s (1980) was the first study to hypothesize that an aphrodisiac pheremone secreted from the male’s head played an important role in *Nasonia* courtship. Until now, the likely source of this pheromone had not been identified, more than 40 yr after the original study was published. Still, the chemical makeup of the aphrodisiac is unknown. The identification and description of the genomandibular gland in *Nasonia* paves the way for future studies to identify the chemical composition of the aphrodisiac phermone.

It has been suspected since the aphrodisiac pheromone was discovered that the source gland should be close to the mandible as, during courtship, males touch the female antenna at the head region between the mandible and the gena (van den Assem et al. 1980, 1981).

The mandibular rods (Bucher 1948, Heraty et al. 2018) were suggested to have glandular function (Ronquist and Nordlander 1989) and were one of the candidate structures as the source of the aphrodisiac pheromone. However, these structures are solid (not hollow) and lack any characteristics of insect exocrine glands, such as cuticular canals, pore canals, or microvilli-rich cellular structures (Locke 1961, Noirot and Quennedey 1974, 1991). Based on the morphology of the adjacent cellular structures with narrow, axon-like projections, the mandibular rods might represent chordotonal organ-like mandibular mechanoreceptors.

We found representatives of the 2 main exocrine gland classes that emptied their extract into the region touching the female antenna. Cells of class I exocrine glands are similar to ordinary epidermal cells as they are adjacent to the cuticle and secrete their extracts through the cuticular pore canals (Locke 1961). On the other hand, class III gland cells are not adjacent to the cuticle, and their secretion is delivered to the cuticle surface via the cuticular canal of a modified epithelial cell, the canal cell (Noirot and Quennedey 1974, 1991). The sites of secretion in both gland classes are characterized by a microvilli-rich surface, which is the distal surface of the cell membrane in class I cells, that faces the cuticle, and the end apparatus in class III gland cells that surrounds the end of the cuticular canal. We were able to locate end apparatuses and cuticular canals only in the genal gland whereas these structures are lacking from both the genomandibular and the intramandibular glands. On the other hand, the apical membrane of these cells is rich in microvilli making it clear that these 2 glands are class I exocrine glands.

The only class III lower head gland in *Nasonia* is the genal gland which has not been reported from any other Hymenopterans. This gland is composed of class III gland cells that empty their extracts through the cuticular canals independently from each other on the inner pleurostomal surface. We did find this gland present in both male and female specimens. Still, similar to the intramandibular gland, we did not find any interspecific or sexual differences between males and females that would correlate to courtship intensity. Ågren (1978) reported the presence of class III gland cells with a similar location on the gena in *Cerceris rybyensis* (Linnaeus) (Hymenoptera: Phylantidae); however, the individual gland ducts of this gland open at the lateral portion of the cranio-mandibular conjunctiva and not through the pleurostoma. The class III mandibular gland is known in multiple aculeate (Hermann et al. 1971, Lensky et al. 1985, Fortunato et al. 2000, Galvani and Settembrini 2013, Penagos-Arévalo et al. 2015, Santos et al. 2015) and 2 proctotrupomorph taxa (Völkl et al. 1994, Stökl and Herzner 2016). These glands, unlike the newly described genal gland that empties at the hypostoma, always empty through a single mandibular gland duct at the base of the mandibular sclerite (Richter et al. 2021). The class I basimandibular gland of *Nasonia* species was also found in other Proctotrupomorpha (Desyatirkina et al. 2023), but has never been properly characterized. Based on its location and the corresponding thinner mandibular cuticle it may be homologous to the basimandibular gland in ants (C. Wang et al. 2021) amongst other known intramandibular glands (Schoeters and Billen 1994, do Amaral and Caetano 2006, Santos et al. 2009, Cruz-Landim et al. 2011, Penagos-Arévalo et al. 2015, Santos et al. 2015, Billen and Al-Khalifa 2016, Billen and Ito 2022). Similarly to the genal gland, we did not find any inter- or intra-specific differences in the dimensions of this gland that would show sexual dimorphism or would correlate to courtship intensity.

The genomandibular gland is composed of modified epithelial cells that correspond to the cuticle at the base of the mandible, the pleurostoma, and the lateral portion of the cranio-mandibular conjunctiva. This gland likewise, represents a so far undescribed gland in Hymenoptera. The modified epithelial cells comprising this gland are characterized by the microvillus-rich distal cell membranes. We have not detected the presence of end apparatuses and cuticular gland canals in the modified epithelial cells or the cells adjacent to them that most likely represent fat body cells. Based on these characteristics, the gland is a class I exocrine gland. The only other gland located in the same position is the malar gland of *Cerceris* (Crabronidae: Cercerini); however, this gland is a class III gland (Ågren 1978).

Our study indirectly shows evidence that the source of the mysterious male aphrodisiac is the genomandibular gland. Amongst the 3 exocrine glands that open at the lower head region, the size of the reservoir (subcuticular space) of only this gland shows a correlation with courtship intensity and sexual dimorphism. In females, the epithelial cells corresponding with the lateral regions of the cranio-mandibular conjunctiva are slightly enlarged, suggesting that a small genomandibular gland may also be present in all female specimens. However, gland volume was not measured due to limitations in delineating the gland in female specimens, and for the purposes of this study is considered to be absent. The gland cells are distinctly larger in *Nasonia vitripennis* males than in females; however, the reservoir (the subcuticular space) is only developed in a small number of male specimens in this species. The reservoir size is larger in *N. longicornis* and *N. oneida*, which share the same size, and the largest in *N. giraulti* males.

The size of the genomandibular gland reservoir corresponds to both genal convexity and the size of the basal mandibular carina. The genomandibular gland in *Nasonia* is a class I gland that does not have cuticular canals to deliver the gland extract, but the gland itself empties through the cuticle through its apical microvilli-embedded membrane region. It is reported that during the time that the gland extract is produced in class I gland cells, a subcuticular space develops, and similar to ecdysis, the epithelial cell layer detaches from the cuticle (Noirot and Quennedey 1974, Quennedey et al. 2002, Costa-Leonardo 2006, Billen and Verbesselt 2016, Billen and Peeters 2020). The subcuticular space often serves as a reservoir, whose size is clearly defined by the size of the detached epithelium and the shape of the underlying cuticular region. Genomandibular gland size may be constrained by head shape. It is possible that the gland cannot grow larger due to spatial interference with the brain, eyes, muscles, etc. The cuticular regions in *Nasonia* males that prune the detached epithelial cell layer are the pleurostoma, the cranio-mandibular conjunctiva, and the base of the mandibular cuticle anterolateral to the site of origin of the mandibular abductor muscles and therefore its area is larger in specimens with larger basal mandibular carina. The basal mandibular carina is adjacent to the pleurostoma and slides along the pleurostomal surface when the mandible is moved. Because the genomandibular gland is a class I gland, the gland extract is most likely released through the corresponding cuticular regions, possibly through the basal mandibular conjunctiva and the surface of the basal mandibular carina. Therefore, it is most likely that the basal mandibular carina serves as a spread and release structure in *Nasonia* species. Its enlarged size not only promotes the production of more gland extract, due to the larger number of involved epithelial cells, but also enhances the efficient release of the extract by increasing the potential evaporative and spread surface.

In most insects with chewing mouthparts, mandibular movement is limited to a single degree of freedom. In Chalcidoidea, however, the mandible lacks a posterior condyle and is loosely articulated with the cranium by a single anterior condyle, which, combined with a transformed mandibular musculature, allows flexible movement in multiple planes (van de Kamp et al. 2022). This unique adaptation likely played an important role in the group’s immense radiation and allowed members of the superfamily to exploit novel host systems. The mandibles of Chalcidoidea have been observed to be used in a chisel-like fashion to bite through stiff substances such as wheat grains (van de Kamp et al. 2022) or used independently to penetrate pliable materials such as insect egg shells (Spiecker et al. 2023). The secondarily monocondylic mandibles of Chalcidoidea and the presumed variable range of motion of the mandible may explain why there is variation in mandibular structure at the family level. As shown here, in addition to biting strategies, mandibular gland systems may benefit from flexible mandibular articulations, and allow for a spread and release structure to function even more effectively. The mandible is the proposed structure for the dispersal and release of the aphrodisiac pheromone for *Nasonia* and may have the same function in other groups (König et al. 2015).

Also investigated as part of this study was the genomandibular gland volume of *Trichomalopsis,* another Pteromalini (Pteromalidae: Pteromalinae) genus. Interestingly, like *Nasonia*, cranium shape varies between species of *Trichomalopsis* (Gibson and Floate 2001). Gibson and Floate (2001) determined that gena convexity is variable at the species level in *Trichomalopsis*. For example, male *Trichomalopsis dubia* (Ashmead) have a more convex gena than male *T. sarcophagae*. Although we did not analyze the gland volume in *T. dubia*, the relatively small subcuticular space in *T. sarcophagae* (being the second smallest after *N. vitripennis* amongst the analyzed taxa) suggests that differences in gena convexity in *Trichomalopsis* may correlate with the mandibular gland in a similar way to what is observed in *Nasonia*. Courtship behavior similar to that observed in *Nasonia* species, including head nodding, has also been reported in *Lariophagus distinguendus* (Förster) (König et al. 2015). An oral gland, akin to that of *Nasonia*, has been identified as the key factor in releasing the aphrodisiac pheromone in this species (König et al. 2015). These findings suggest that the genomandibular gland is more broadly distributed within the Pteromalidae family, and its influence on cranial morphology provides a valuable character for species-level identification in this group.

More broadly, within Chalcidoidea there are other examples of pheromone spread and release structures varying on a species level (Mitroiu 2010, Shirley et al. 2019). *Aphelinus* Dalman (Aphelinidae) is a genus of parasitoid wasps that may release a pheromone during courtship via the pores on male scapes (Shirley et al. 2019). Shirley et al. (2019) investigated several scape characteristics and found that combinations of a selection of these traits are diagnostic for species complexes, and in some cases within species complexes as well. The present study found species-level variation in what is hypothesized to be the pheromone spread and release structure in *Nasonia*, as the surface area of the cranium invagination adjacent to the mandible varies between species of *Nasonia*. Our study indicates that variations in pheromone spread and release structures may be catalysts for speciation in Chalcidoidea.

*N. vitripennis*, *N. longicornis,* and *N. giraulti* have distinct differences in the morphology of antennal scape and wing length (Darling and Werren 1990) and the convexity of the male gena (Werren et al. 2016, Cohen et al. 2021). The fourth *Nasonia* species, *N. oneida* is considered the sister species to the sympatric *Nasonia giraulti* and although it is distinct from *N. giraulti* based on molecular markers, cuticular hydrocarbon profiles, and behavioral characteristics, the 2 species share similar antennal and wing morphology and differ only in subtle morphological differences (Raychoudhury et al. 2010). Surprisingly, both the genal convexity and gland/reservoir morphology of *N. oneida* were most similar to *N. longicornis* and not *N. giraulti*.

The present study found that genomandibular gland volume is smallest in *N. vitripennis*, and largest in *N. giraulti*, with *N. longicornis* and *N. oneida* having an intermediate volume. Genomandibular gland size is correlated to increased gena convexity in *Nasonia* males; *N. giraulti* has the most convex gena while *N. vitripennis* has the least convex gena, while *N. longicornis* and *N. oneida* have intermediate gena convexity.

These findings inspired the question of the relationship between courtship cycle length and genomandibular gland size. *N. giraulti* may have the longest courtship cycle simply because their genomandibular gland can hold more aphrodisiac pheromone, which can then be released over a longer period, or in larger pulses. It may be that the genomandibular gland cells in *N. giraulti* are not *producing* more pheromone, but rather can *store* more. On the other hand, *N. vitripennis* does not have a gland reservoir and instead may use a “pay as you go” mechanism for producing and releasing gland extract; *N. vitripennis* is reliant on the amount of gland extract that can be produced on demand for their courtship. Differences in gland volume exhibit a direct correlation to courtship cycle length and gena convexity, showing a clear link between structure and function and explaining why certain species can have a longer courtship cycle than others. Additionally, *N. giraulti* males can induce 100% female receptivity with less head nodding series (van den Assem and Werren 1994). Therefore, it may be adaptive for *N. giraulti* to have a larger genomandibular gland and a longer courtship cycle, thus requiring fewer cycles. It is possible this is because there is a great energetic cost for *N. giraulti* to start a cycle of courtship. *N. vitripennis* is hypothesized to be proficient in gland extract production “on the fly” due to the “pay as you go” mechanism. Because courtship *in N. vitripennis* occurs outside the host, with virgin females rapidly emerging, males must court quickly before females disperse or are courted by a different male. In addition, males must determine quickly whether the female is receptive (already mated females will not accept the male, and males can quickly give up and move on to other opportunities, (Ruther et al. 2007). Male interference in courtship occurs, and this is another advantage of rapid courtship cycles.

Female receptivity correlates with head nods and mouthpart extrusion suggesting that pheromone discharge occurs at this time (van den Assem and Werren 1994). *Nasonia* species mate either within the host puparium (WHM), or outside the puparium, directly after the female emerges where the male awaits (Drapeau and Werren 1999, Trienens et al. 2021). Drapeau and Werren (1999) found the highest levels of WHM in *N. giraulti*, intermediate in *N. longicornis*, and rare in *N. vitripennis*. Trienens et al. (2021) found a similar pattern but also evaluated that *N. oneida* has an intermediate amount of WHM. Notably, the proportion of WHM has a direct correlation to *Nasonia* genomandibular gland size and gena convexity and courtship cycle length; the greater proportion of WHM in a species, the larger the genomandibular gland and the longer the courtship cycle. The fact that *N. giraulti* exhibits the greatest proportion of WHM may explain why the species has the longest courtship cycle. Mating leaves the wasps in a vulnerable state, and having a longer courtship cycle means they are more vulnerable to predation. However, mating *inside* the puparium offers a degree of protection from predation, and may allow for the *N. giraulti* courtship cycle to last longer. Another possible explanation for the larger glands in WHM species is that background pheromone levels build up within hosts, requiring a larger bolus of pheromone to be detected by the female.

Cohen et al. (2021) found the same pattern of cheek convexity as in the present study; *N. vitripennis* has the least convex cheeks, *N. giraulti* has the most convex cheeks, and *N. longicornis* has intermediate cheek convexity. Cohen et al. (2021) speculated the differences in convexity were attributed to underlying structures, such as a mandibular gland. The present study builds on this research and shows that there is a correlation between increasing genomandibular gland size and cheek convexity which aligns with the results from Cohen et al. 2021. The *doublesex* gene was investigated by Cohen et al. (2021) as the cause of the differences in cheek convexity, as *doublesex* is observed to cause developmental differences in sex-specific traits between species. Cohen et al. (2021) found that *doublesex* plays a role in creating the male *N. giraulti* head shape. It is therefore likely that this gene is specifically affecting the morphology of the underlying genomandibular gland in *Nasonia,* which in turn creates the respective cheek convexity. Our findings provide a link between the long-observed correlation of gena shape and behavior. Recent exploration of the impact of the *doublesex* gene on head shape and the effectiveness of the aphrodisiac pheromone have revealed that manipulating *doublesex* negatively impacts both systems, as *doublesex* mutant *N. vitripennis* males were not able to elicit receptivity in conspecific females (Wang et al. 2022), and on the other hand, *N. giraulti doublesex* mutants had substantially smaller cheek size (Cohen et al. 2021). Cohen et al. (2021) observed the effect of the *doublesex* gene on head shape, while Wang et al. (2022) describes the effect of *doublesex* on the ability to attract and induce receptivity in females. The findings of the present study can link together previous work regarding *Nasonia.* This study demonstrates that cheek convexity directly impacts the size of the reservoir of the class I genomandibular gland; *Nasonia* head shape may be directly related to the amount of aphrodisiac pheromone stored.

Congenital craniofacial anomalies are among the most common birth defects in humans (Salari et al. 2022). Occurring in roughly one in every 3000 live births (Salari et al. 2022), abnormalities such as oral clefts have complex genetic origins involving many genes and epistatic interactions, as can be predicted from genome-wide association studies (He et al. 2020, Mukhopadhyay et al. 2022). For several reasons, insect models provide an important reference for studying craniofacial abnormalities in humans (Werren et al. 2016). For one, bilaterally symmetric animals share cephalization as an ancestral trait, so there is a basis for comparison. Secondly, an analogy can be drawn between the formation of the human cranial case incorporating alimentary and sensory structures and the formation of a cohesive structure in insects from the combination of various tissue types and imaginal discs (Werren et al. 2016). As craniofacial anomalies in both *Nasonia* and *Homo sapiens* are explained by epistatic interactions, understanding the intricacies of these interactions in *Nasonia* will be advantageous for understanding them in humans. This study provides another trait for QTL analysis (genomandibular gland size) which may help elucidate the genetic origins of craniofacial anomalies. The study also provides a link between internal and external cranial structure, as the relationship between head shape variation and internal structure was previously unknown (Werren et al. 2016).

Mandibular gland volume in *Nasonia* varies interspecifically between males and correlates to patterns of courtship behavior such as courtship cycle length and tendency toward within-host mating. This evidence supports the hypothesis that the genomandibular gland is a source of aphrodisiac pheromone. Its development is likely influenced by the *doublesex* gene. Further investigation of this phenomenon will allow for an increased understanding of insect chemical ecology and the relationship between evolution and development.

## Supplementary Material

ieaf034_suppl_Supplementary_Tables_1

ieaf034_suppl_Supplementary_Figures_1
