# Supplementary material for: The cranial gland system of Nasonia spp.: a link between chemical ecology, evo-devo, and descriptive taxonomy (Hymenoptera: Chalcidoidea)
Source: J Insect Sci. 2025 Apr 15;25(2):13. doi: 10.1093/jisesa/ieaf034 (PMC11997971; doi:10.1093/jisesa/ieaf034)
Supplement: ieaf034_suppl_Supplementary_Tables_1 [file ieaf034_suppl_supplementary_tables_1.pdf]

Label	Concept	URI	Preferred Term		
adductor muscle	The mandibular muscle that arises posterodorsally from the cranium and inserts on the tendon attached anteroproxim	http://purl.obol	posterior cranio-mandibular muscle		
adductor muscle	The mandibular muscle that arises posterodorsally from the cranium and inserts on the tendon attached anteroproxim	http://purl.obol	anterior cranio-mandibular muscle		
anterior adductor	The adductor muscle that inserts on the mandible anteriorly.	http://purl.obol	anterior adductor		
anterior angle of mandible	The edge of the anterior mandibular surface that corresponds to a notch anteriorly on the pleurostoma.	http://purl.obol	anterior angle of mandible		
anterior view	An anatomical region that is the entire part of an anatomical structure anterior to a transverse plane and bounded on	http://purl.obol	anterior side		
articulation	The region of cuticle that is composed two articular surfaces that are adjacent to each other.	http://purl.obol	insect articulation		
basal mandibular carina	The transverse carina at the base of the mandible just poroximally of the basal mandibualr connectiva	http://purl.obol	basal mandibular carina		
basal mandibular conjunctiva	The conjunctiva that encircles the proximl margin of the mandible.	http://purl.obol	basal mandibular conjunctiva		
basimandibular gland	The class I exocrine gland that is on the posterior (ventral) edge of the mandible and that corresponds to a thin cuticul	http://purl.obol	basimandibular gland		
BCH, basal mandibular carina	The anatomical line that is perpendicular to the basal line of the basal mandibular carina and extends between the ba	http://purl.obol	basal mandibular carina		
carina	The process that is elongate and external.	http://purl.oboli	carina		
cell	A material entity of anatomical origin (part of or deriving from an organism) that has as its parts a maximally connecte	http://purl.obol	cell		
class I gland	The exocrine gland that is composed of epithelial cells that has a microvilli rich apical region.	http://purl.obol	class I gland cell		
class III gland cell	The exocrine gland that is composed of epithelial cells that encircle cuticular endocrine gland ducts that ends with an	http://purl.obol	class III gland cell		
conjunctiva	The area of the cuticle that is more flexible than adjacent sclerites.	http://purl.oboli	conjunctiva		
convex	A complete three dimensional shape in which for every line connecting pair of points on the object is within the object.	http://purl.obol	convex		
cranium	The sclerite that is articulated with the cervical prominence, the scapes and the mandibles.	http://purl.oboli	cranium		
cuticle	The acellular anatomical structure that is the external layer of the integument (covers the entire body surface as well as lines ectoc	http://purl.oboli	cuticle		
cuticle embedded ducts	The branching cuticular invagination that corresponds to the invagination of the membrane of a single cell and does n	http://purl.obol	cuticular exocrine gland duct		
cuticular pores	The orifice that is contained by the cuticle and connects the inside of the insect with the outside, is tubular, and whose	http://purl.obol	cuticular pore		
cytoplasm	The contents of a cell excluding the plasma membrane and nucleus, but including other subcellular structures.	http://purl.obol	cytoplasm		
electron dense	A color brightness which is relatively low.	http://purl.obol	low brightness		
electron lucent	A color brightness which is relatively high.	http://purl.obol	high brightness		
end apparatus	The microvilli rich chamber located at the distal portion of cuticular exocrine gland duct.	http://purl.obol	end apparatus		
pleurostoma	The area that extends on the ventral (anterior) margin of the cranium along the site of origin of the conjunctiva connec	http://purl.obol	pleurostoma		
epithelial cell	A cell that is usually found in a two-dimensional sheet with a free surface. The cell has a cytoskeleton that allows for ti	http://purl.obol	epithelial cell		
epithelium	Portion of tissue, that consists of one or more layers of epithelial cells connected to each other by cell junctions and w	http://purl.obol	epithelium		
foramen	The anatomical space that is surrounded by sclerites and allows for the passage of haemolymph, nerves and tracheae.	http://purl.oboli	foramen		
gena	The area that is delimited by the intersection of the interorbital plane, the margin of the compound eye, the margin of the oral fora	http://purl.oboli	gena		
genal gland	The class III exocrine gland that is located inside the gena and empties on the lateral portion of the pleurostoma.	http://purl.obol	genal gland		
genomandibular gland	The class I exocrine gland that corresponds to the basal mandibular conjunctival region between the lateral portion of	http://purl.obol	genomandibular gland		
GH	The anatomical line that is longest perpendicular line connecting the genal width and the ventrolateral amrgin of the g	http://purl.obol	gena height		
gland cell	A specialized epithelial cell that is capable of synthesizing and secreting certain biomolecules.	http://purl.oboli	gland cell		
GW	The anatomical line that connects distal median point of the clypeus and the diorsalmost point of the compound eye ir	http://purl.obo	gena width		
HW	The anatomical line that is the longest horizontal diameter of the cranium in frontal view.	http://purl.obol	head width		
lateral abductor	The posterior cranio-mandibular muscle that inserts on the mandible laterally	http://purl.obol	lateral abductor		
mandible	The appendage that is encircled by one sclerite that is connected to the cranium proximolaterally and to the maxillo-labial comple	http://purl.oboli	mandible		
mandibular foramen	The foramen that is located proximally on the mandible.	http://purl.obo	mandibular foramen		
mandibular rod	The sclerotized, rodlike solid invagination of the mandibular tooth that extends almost along the entire length of the to	http://purl.obo	mandibular rod		
margin	The line that delimits the periphery of an area.	http://purl.oboli	margin		
median abductor	The posterior cranio-mandibular muscle that inserts on the mandible laterally	http://purl.obol	median abductor		
microvilli rich apical region	A layer of microvilli, Thin cylindrical membrane-covered projections on the surface of an animal cell containing a core	http://purl.obol	microvilli rich apical region		
muscle	Muscle tissue that consists primarily of skeletal muscle fibers.	http://purl.obol	skeletal muscle		
oral foramen	The foramen that is located anteriorly/ventrally on the cranium in which the mouthparts are positioned.	http://purl.oboli	oral foramen		
posterior adductor	The adductor muscle that inserts on the mandible posteriorly.	http://purl.obol	posterior aductor		
posterior angle of mandible	The edge of the anterior mandibular surface that corresponds to a notch posteriorly on the pleurostoma.	http://purl.obol	posterior angle of mandible		
rim	The carina that extends along the margin or edge of a sclerite.	http://purl.oboli	rim		
sclerite	The area of the cuticle that is less flexible than adjacent conjunctivae.	http://purl.oboli	sclerite		
structure	Material anatomical entity that is a single connected structure with inherent 3D shape generated by coordinated expression of the	http://purl.obol	anatomical structure		
subcuticular space	The anatomical space between the microvilli rich apical region of the class I exocrine gland cells and the cuticle.	http://purl.obol	subcuticular space		
surface	Material anatomical entity that forms the outermost boundary of an anatomical structure.	http://purl.obol	anatomical surface		
tooth	The projection that is located distally on the mandible.	http://purl.obol	tooth		
vesicles	Any small, fluid-filled, spherical organelle enclosed by membrane.	http://purl.obol	vesicle		
volume	A 3-D extent quality inhering in a bearer by virtue of the bearer's amount of 3-dimensional space it occupies.	http://purl.obol	volume		
