# Supplementary figures and images for: The cranial gland system of Nasonia spp.: a link between chemical ecology, evo-devo, and descriptive taxonomy (Hymenoptera: Chalcidoidea)
Source: J Insect Sci. 2025 Apr 15;25(2):13. doi: 10.1093/jisesa/ieaf034 (PMC11997971; doi:10.1093/jisesa/ieaf034)

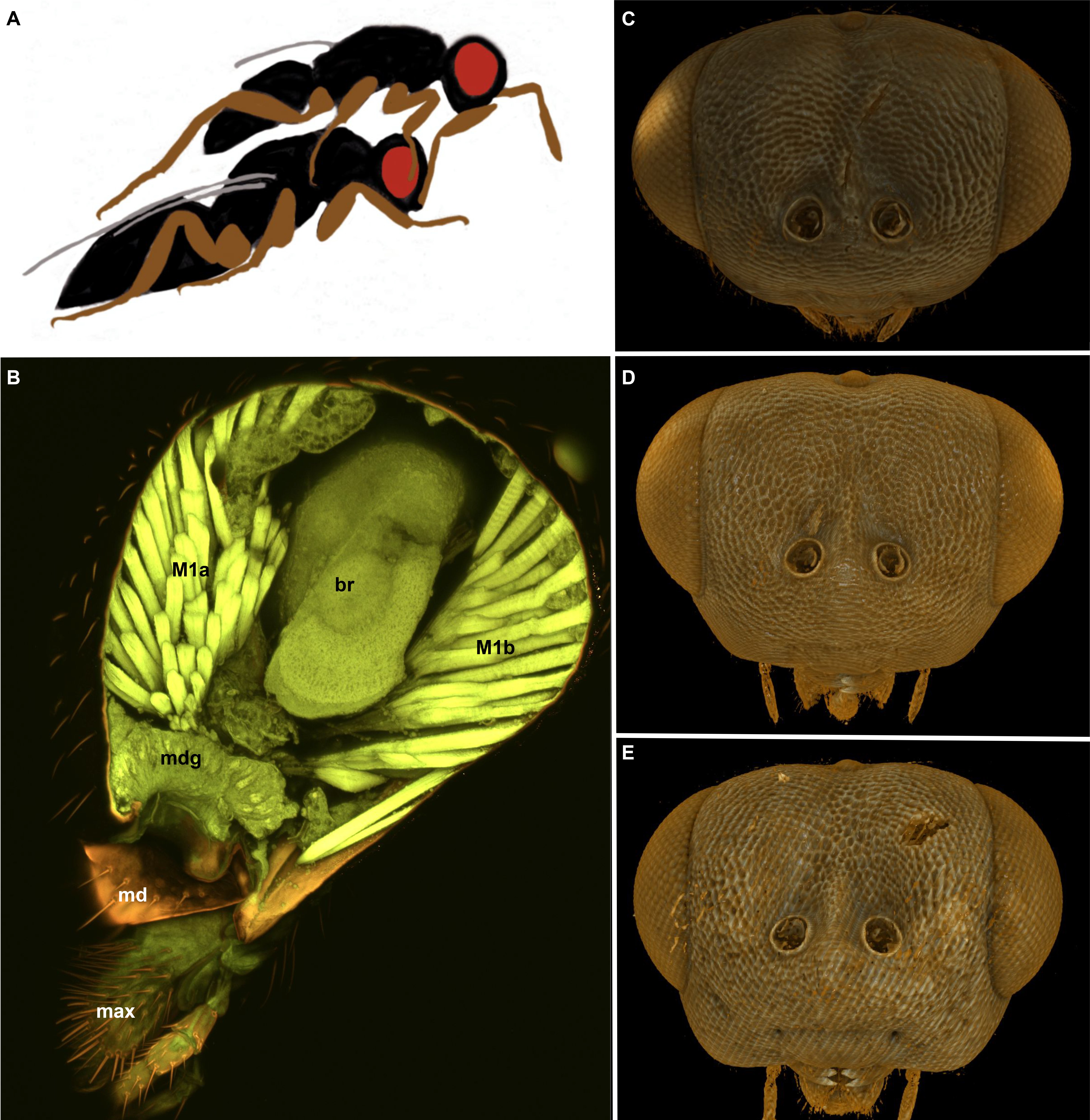

Supplement: ieaf034_suppl_Supplementary_Figures_1 [file ieaf034_suppl_supplementary_figures_1.jpeg]
